# IgA-Type Enterovirus Antibodies Are Increased among Adults and Children with Recently Diagnosed Type 1 Diabetes

**DOI:** 10.1155/2022/7603062

**Published:** 2022-07-31

**Authors:** Kristi Alnek, Ija Talja, Brita Laht, Kaja Metsküla, Maire Mandel, Ingrid Reppo, Maire Lubi, Raivo Uibo

**Affiliations:** ^1^Department of Immunology, Institute of Bio- and Translational Medicine, University of Tartu, 19 Ravila, Tartu 50411, Estonia; ^2^Department of Internal Medicine, Institute of Clinical Medicine, University of Tartu, 8 L. Puusepa, Tartu 50406, Estonia; ^3^Internal Medicine Clinic of Tartu University Hospital, 8 L. Puusepa, Tartu 50406, Estonia

## Abstract

Enteroviruses (EV) are among the leading environmental triggers of childhood-onset type 1 diabetes (T1D). Our aim was to determine the prevalence of antibodies against EV and their association with T1D in different age groups (*n* = 62), including young adults, and to compare these data with results from HLA-matched control participants (*n* = 62). IgA, IgG, and IgM antibodies against EV were detected. IgA EV antibodies were present in 46.8% of participants with T1D (median level 10.9 EIU) and in 11.3% of controls (median level 3.4 EIU). IgA EV positivity and higher level of IgA EV antibodies were both significant risk factors for T1D (odds ratio (OR) 8.33; 95% confidence interval (CI) 2.52–27.6; *p* = 0.0005 and OR 1.04; 95% CI 1.01–1.06; *p* = 0.0105, respectively). Importantly, the prevalence of IgA EV antibodies in the subgroups of both children and young adults was also significantly different between participants with T1D and their matched controls (*p* = 0.0089 and *p* = 0.0055, respectively). Such differences were not seen for IgG and IgM EV antibodies. However, IgG EV antibodies were associated with 65 kDa glutamic acid decarboxylase antibodies, but not with zinc transporter 8 and protein tyrosine phosphatase IA2 antibodies. The genotype frequency of PTPN22 (rs2476601) and IFIH1 (rs1990760) was not associated with EV positivity. This study showed that EV infections may be an important disease-promoting factor of T1D not only in childhood-onset but also in adult-onset T1D. However, to further confirm this association, direct virological studies are needed in the latter T1D group.

## 1. Introduction

Type 1 diabetes (T1D) is a chronic immune-mediated disorder characterized by progressive destruction of insulin-producing *β*-cells. The major genetic risk for T1D is related to the human leukocyte antigen (HLA) complex, where the combination of HLA class II alleles DRB1∗03/DQB1∗0201 and DRB1∗04/DQB1∗0302 confers the greatest risk [1]. However, a previous Finnish study has shown that this high risk genotype was only detected in 21% of T1D cases [2]. It has also been suggested that environmental factors such as low vitamin intake, cow's milk exposure, breastfeeding, toxins, high birth weight, gut microbiome, and viruses are triggers for onset of the disease [1, 3].

Enteroviruses (EV) are considered the leading candidates according to numerous studies performed in children [4–6]. The association of EV with T1D has been confirmed both by the presence of EV protein in the pancreatic tissue [7] and by serum antibodies to EV in T1D [8, 9]. However, it is not fully known whether T1D in adults is associated with EV. There are no sufficient data about the role of other T1D susceptibility genes in the modulation of EV infections among the adult population. In this context, interferon induced with helicase C domain 1 (IFIH1) protein, a sensor for EV infection [5, 10], and protein tyrosine phosphatase nonreceptor type 22 (PTPN22), a significant modulator of immune response [5, 11], deserve special attention. The role of both genes and their polymorphisms has received significant attention in connection with T1D in recent years [12].

These genetic and environmental factors can lead to early-stage T1D where the first islet autoantibodies (AAB) develop (stage 1) [13]. The main AAB are against insulin, 65 kDa glutamic acid decarboxylase (GADA), protein tyrosine phosphatase (IA2A), and zinc transporter 8 (ZnT8A) [14]. All these autoantibodies indicate the presence of autoimmune reactions against *β*-cells of the pancreas. In the further disease process, dysglycaemia develops at stage 2 and clinical symptoms manifest themselves at stage 3 [13].

In this study, we aimed to determine the prevalence of antibodies against EV in participants with clinically diagnosed stage 3 T1D and in HLA-matched controls in different age groups. We also aimed to determine the distribution of PTPN22 and IFIH1 genotypes and autoimmune characteristics in T1D cases with and without EV antibodies, as well as to reveal their association with EV antibody positivity.

## 2. Materials and Methods

### 2.1. Study Population

Participants with clinically diagnosed stage 3 T1D (*n* = 62) and matched control participants (*n* = 62) <35 years of age were included in this nested case-control study (Table 1). Participants with T1D and controls were randomly pair-matched by age, gender, and HLA-DR/DQ genotype risk groups. The median age difference between participants with T1D and matched controls was 0.4 years (range: 0.0–4.1).

Adult participants with T1D were recruited from the Internal Medicine Clinic of Tartu University Hospital. Children and adolescents with T1D were seen at the Children's Clinic of Tartu University Hospital or at Tallinn Children's Hospital. In all participants, the diagnosis of T1D was based on internationally approved diagnosis criteria [15]. Peripheral blood samples were obtained on the day of diagnosis or within 17 days of diagnosis. Random C-peptide values were in the range 0.04 to 1.10 nmol/L (mean 0.22 nmol/L, reference range = 0.37–1.47 nmol/L). Of the participants with T1D, 38.7% had ketoacidosis (Table 1). All participants with T1D were receiving insulin treatment during the time of sample collection. Data about self-reported concomitant autoimmune diseases was collected: autoimmune thyroiditis was recorded in seven, vitiligo in one, and rheumatic diseases in one participant with T1D. None of the individuals participating in this study had a history of infections during the past month, except for two participants with T1D who had had respiratory infections.

The control group consisted of children and adults without diabetes, who visited Tartu University Hospital with minor surgical indications, and volunteers from among the laboratory and university personnel. The controls for this study were selected synchronously with recruitment of participants with T1D. All control participants had normal blood glucose or glycated haemoglobin (HbA1c) level and were negative for AAB. All participants with T1D and controls (parents or guardians for children) signed a written consent form before participation in the study. All participants for this study were recruited between March 2008 and September 2018. Approval from the Research Ethics Committee of the University of Tartu (Estonia) was obtained (protocols 163/T-6 from 24.09.2007 and 275/M/15 from 20.11.2017). One adult control participant had autoimmune thyroiditis, and two control participants had rheumatic diseases.

### 2.2. HLA Genotyping

HLA-DR/DQ genotyping was performed by polymerase chain reaction- (PCR-) based lanthanide-labelled oligonucleotide hybridization and by a time-resolved fluorescence assay as described elsewhere [2, 16, 17]. Based on data about HLA-DR/DQ haplotype subgrouping by Ilonen et al. [2], we distributed our participants with T1D and controls into groups with increased, neutral, and decreased risk for T1D. There were not enough study subjects for further grouping, as was done in the original study.

### 2.3. Antibodies against Enteroviruses

The presence of IgA and IgG antibodies to EV was tested by using an enzyme-linked immunosorbent assay (ELISA). For the antigen, the common synthetic EV peptide KEVPALTAVETGAT (Storkbio Ltd., Estonia) was employed, as described elsewhere [9]. The test results were expressed in enzyme immunoassay units (EIU) calculated with the formula: [(sample OD–negative reference OD)/(positive reference OD–negative reference OD)] × 100, where OD means optical density. Test values ≥ 15 EIU were defined as a positive result against EV antibodies, according to the results of previous studies [9].

IgM-type antibodies to EV were evaluated employing the commercial ELISA kit according to the manufacturer's instructions (Euroimmun Medizinische Labordiagnostika AG, Lübeck, DE), using recombinant VP1 antigens from coxsackievirus and echovirus. Altogether, 57 serum pairs from controls and participants with T1D were studied, since for 5 pairs, serum samples were exhausted. The semiquantitative test results were calculated with the following formula: ratio = control or sample OD/calibrator OD. The results were interpreted as follows: ratio ≥ 1.1 is positive.

### 2.4. Islet Autoantibodies

Antibodies against GADA, IA2A, and ZnT8A were measured using commercial ELISA kits according to the manufacturer's instructions (RSR Ltd., Cardiff, UK). The cut-off level for positivity was the following: ≥5 U/mL for GADA, ≥15 U/mL (tests up to May 2015) or ≥7.5 U/mL (tests after May 2015) for IA2A, and ≥15 U/mL for ZnT8A. These tests have been under regular external quality control by the Islet Autoantibody Standardization Program [18].

### 2.5. PTPN22 and IFIH1 Polymorphisms

PTPN22 (rs2476601) and IFIH1 (rs1990760) polymorphisms were determined by the TaqMan single-nucleotide polymorphism genotyping assay (Applied Biosystems, Thermo Fisher Scientific, USA) (assay ID: C__16021387_20 and C__2780299_30, respectively) [19].

### 2.6. Statistical Analyses

Data analyses were performed in the R version 3.6.1 (Free Software Foundation, Boston, MA), and figures were prepared in GraphPad Prism 5 (GraphPad Software, La Jolla, CA). The PTPN22 and IFIH1 Hardy-Weinberg equilibrium was verified with the chi-square test, without evidence for deviation. Differences between the participant groups and categorical characteristics were calculated with the chi-square or Fisher's exact test. Differences between diagnosis and nonparametric characteristics were calculated with the Mann-Whitney *U* test (two-tailed). Relationship between diagnosis and EV antibodies was analysed using a conditional logistic regression model. Matched odds ratios (OR) for gender, age, and HLA-DR/DQ genotype risk groups for T1D with the 95% confidence interval (CI) were calculated. The Cochran-Mantel-Haenszel test was used to analyse associations between diagnosis and EV antibodies for different age groups; *p* values were calculated. In further analysis, participants with T1D and controls were evaluated separately. Diagnosis-stratified multiple logistic regression analysis, adjusted for age, gender, and HLA risk, was used to find associations between the presence of EV antibodies and characteristics presented in Table 1. Adjusted odds ratios (adOR) and 95% CI were calculated. The Epi package in R was used to calculate the area under the curve (AUC). For diagnosis-stratified multiple linear regression analysis, EV antibody levels were log2 transmitted. Multiple linear regression analysis, adjusted for age, gender, and HLA risk, was used to find associations between the EV antibody level and characteristics presented in Table 1. Characteristics and model *p* values and model adjusted *R*-squared (ad*R*^2^) were calculated. *p* values ≤ 0.05 were considered statistically significant.

## 3. Results

### 3.1. Prevalence of Antibodies against Enterovirus

**Figure 1:**
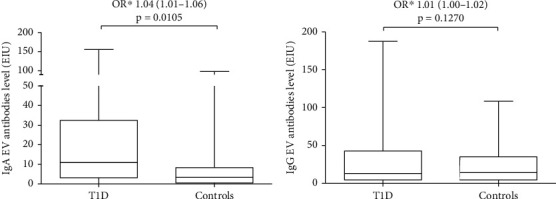
Differences in (a) IgA and (b) IgG enterovirus (EV) antibody levels between participants with T1D and controls. ^∗^Conditional logistic regression model: matched odds ratio for gender, age, and HLA-DR/DQ genotype risk groups for T1D with the 95% confidence interval (CI). *p* values ≤ 0.05 were considered significant.

Twenty-nine of the 62 participants with T1D (46.8%; 95% confidence interval (CI): 34.2–59.8) and 7 of the 62 control participants (11.3%; 95% CI: 5.0–22.5) were positive for IgA EV antibodies (Table 2). Median IgA EV antibody level was 3.4 EIU in the control group (IQR: 0.5–8.1) and 10.9 EIU (IQR: 3.7–31.7) in the T1D group. Thus, IgA EV antibody positivity had strong significant association with T1D (odds ratio (OR) 8.33; 95% CI: 2.52–27.6; *p* = 0.0005) (Table 2). Importantly, participants with T1D also had significantly higher IgA EV antibody levels (OR 1.04; 95% CI: 1.01–1.06; *p* = 0.0105) (Figure 1(a)).

IgG EV antibodies were present in 30 participants with T1D (48.4%; 95% CI: 35.7–61.3) and in 29 controls (46.8%; 95% CI: 34.2–59.8) (Table 2). Median IgG EV antibody level was 14.3 EIU (IQR: 4.9–33.4) in the control group and 12.6 EIU (IQR: 4.7–41.5) in the T1D group. There were no statistically significant differences in IgG EV antibody positivity or antibody levels between the T1D and the control groups (Table 2, Figure 1(b)).

Three out of 57 participants with T1D (5.2%; 95% CI: 1.4–15.5) and one control participant (1.8%; 95% CI: 0.09–10.6) were positive for IgM EV antibodies (Table 2). None of the IgM EV antibody-positive persons were positive for the IgA EV antibody. At the same time, two out of three IgM EV antibody-positive participants with T1D were also positive for IgG EV antibodies.

### 3.2. Prevalence of Antibodies against Enterovirus in Different Age Groups

**Figure 2:**
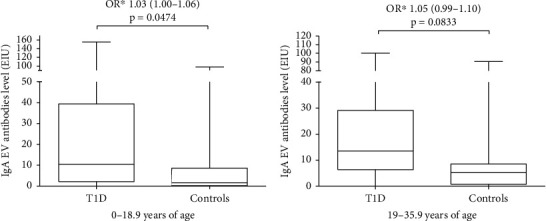
Differences in IgA enterovirus (EV) antibody level between participants with T1D and controls by the age groups (a) 0-18.9 years of age and (b) 19-35.9 years of age. ^∗^Conditional logistic regression model: matched odds ratio for gender, age, and HLA-DR/DQ genotype risk groups for T1D with the 95% confidence interval (CI). *p* values ≤ 0.05 were considered significant.

Prevalence of antibodies to EV in different age groups is presented in Table 3. The prevalence of IgA EV antibodies was significantly higher in paediatric participants with T1D (0–18.9 years of age) (*p* = 0.0098) compared to control participants of the same age. Interestingly, a similar difference in IgA EV antibody positivity was seen in the adult study group (19–35.9 years of age) (*p* = 0.0055). There was no difference in IgG or IgM EV antibody positivity between T1D and control in either age group. Also, there was a significant difference in IgA EV antibody levels between T1D and control among children (OR 1.03; 95% CI: 1.00–1.06, *p* = 0.0474) (Figure 2(a)). Among adults, only a trend for higher IgA EV levels was seen in T1D compared to control (Figure 2(b)).

### 3.3. Factors Affecting Enterovirus Antibodies in Participants with T1D

Multiple logistic regression analysis adjusted for age, gender, and HLA genotypes revealed that the likelihood of participants with T1D to have IgA EV antibodies tended to be increased by female gender (adOR 4.70, 95% CI: 1.43-15.45, *p* = 0.0107). The AUC for this model was 0.781. In the multiple linear regression model adjusted for age and gender, IgA EV antibody level tended to be increased by (i) female gender (*p* = 0.0021) compared with male gender and (ii) GADA positivity (*p* = 0.0003) compared with GADA negativity (model ad*R*^2^ = 0.3061, *p* = 2.14*e* − 05). In the linear regression model adjusted for age, gender, and HLA genotype, IgA EV level tended to be increased by (i) ≥2 AAB (*p* = 0.0031) compared with AAB negative and single AAB positive, (ii) age (*p* = 0.0069), and (iii) female gender (*p* = 0.0031). The ad*R*^2^ for the model was 0.233 (*p* = 0.0012). IgA EV antibody positivity and IgA EV antibody level were not associated with genotypes, IA2A, ZnT8A, or any other variable presented in Table 1 (data not shown). Table 1 displays a similar distribution of PTPN22 and IFIH1 SNPs between the participants with T1D and the control participants. Median IgA EV levels among AAB are presented in Supplementary Table 1.

For the group of participants with T1D, the logistic regression model, adjusted for age, gender, and HLA genotype, revealed that IgA EV antibody positivity increased the risk for IgG EV antibody positivity (adOR 3.87; 95% CI 1.18-12.7, *p* = 0.0254). This model also showed a trend for risk increase with age (adOR 1.06; 95% CI 1.00-1.13, *p* = 0.0554). The AUC for this model was 0.732. In the linear regression model, adjusted for age, gender, and HLA genotype, IgG EV antibody level tended to be increased with (i) age (*p* = 0.0079) and (ii) ZnT8A positivity (*p* = 0.0225) compared with ZnT8A negativity (model ad*R*^2^ = 0.177, *p* = 0.0066). The presence of IgG EV antibodies or IgG EV antibody level was not associated with genotypes, GADA, IA2A, multiple AAB positivity, or any other variable presented in Table 1 (data not shown). Median IgG EV levels among AAB are presented in Supplementary Table 1.

### 3.4. Factors Affecting Enterovirus Antibodies in Controls

For the group of control participants, there was no significant association with IgA or IgG EV antibody positivity in the logistic regression model. IgA EV antibody level tended to be increased in association with IgG EV antibody level (*p* = 0.0021) (model ad*R*^2^ = 0.1671, *p* = 0.0087).

## 4. Discussion

The results of the present study support earlier investigations reporting association between enterovirus infections and T1D. We confirmed previous EV infection in the studied subjects by testing IgA and IgG antibodies against a synthetic EV peptide which contains an epitope sequence common to a number of known EV [20]. Recent contacts with EV have been evaluated by the presence of IgM EV antibodies. To exclude the influence of the most important confounding factors like gender, age, and HLA, participants with T1D and controls in the present study were matched by these variates.

As the most important result, we found that, like childhood-onset T1D, adulthood-onset T1D is associated with EV infection. Hence, in both children and adults, IgA EV antibodies were found more commonly in participants with T1D compared to their age-, gender-, and HLA-matched controls. In addition, our results confirmed the findings of previous studies that paediatric participants with T1D are more frequently IgA EV antibody positive than their peers of the control group [21]. IgG EV antibodies as markers for earlier infections or/and polio vaccinations were detected at similar percentages in the disease and control groups, as shown previously [22–24]. In the present study, IgM EV antibodies were only detected in four subjects, including three participants with T1D. This low prevalence of IgM EV antibodies in our material might indicate that acute EV infections in our study groups were rare at the time of the study.

We showed that participants with T1D had significantly higher blood IgA EV level, while their IgG EV level was similar to that of controls. Similarly, Imagawa et al. [25] showed higher IgA EV level in participants with T1D compared with controls. At the same time, their study participants with autoimmune T1D had lower IgG EV level compared with controls. One of the main reasons for the different results might be connected with genetic differences between the studied populations, as the study by Imagawa et al. is based on an Asian population, while our study material originates from a European ancestry. It should be noted that IgA and IgG EV levels have not been systematically studied in European adult populations. The association of EV infection with T1D has been shown to depend on the continent, detection method, and source of a sample [26].

These findings demonstrate an association between EV IgA antibodies and adult-onset T1D which may support the hypothesis that EV infection is an aetiologic risk factor for T1D not only among children but also among young adults. Of course, the immunopathological process of T1D starts months (or even years) before the actual diagnosis can be made based on clinical symptoms and blood sugar level increase [13]. But the higher prevalence of EV IgA antibodies in the group of participants with T1D lends support to the importance of mucosa-related EV-mediated immune processes, as most IgA-secreting plasma cells are localized in mucosal tissues, foremost the gut [27], where EVs are mostly detected during the infection. Because there was no difference in IgG antibody prevalence between the T1D and control cases, we propose that both participants with T1D and controls had had similar exposure to earlier IgG antibody-inducing EV contacts [24]. On the contrary, IgA EV antibodies may indicate a recent or lasting mucosal infection [3]. Mucosal EV infection can lead to functional and structural changes resulting in islet autoimmunity development [3]. Therefore, we can conclude that the difference in IgA seroprevalence in the adult group suggests that EV infection might be similarly involved in adulthood-onset T1D development, as it triggers childhood-onset T1D. Which of the known EV strains is involved in it remains open. Because there are several candidate EV strains for the aetiology of T1D [3, 28, 29], our approach to use a common peptide antigen to assess recent EV exposure is the most appropriate option. Of course, to confirm the association between EV and adult-onset T1D, viral RNA, and protein, such as VP1, investigations of the pancreatic tissue should be performed in cases of recently diagnosed adulthood diabetes. Serum EV RNA is detectable only during a rather narrow timeframe during EV infection [30] and would hence be a poor analyte in studies evaluating associations between EV and T1D.

There were significantly more IgA EV-positive subjects among female participants with T1D than among male participants with T1D. Although earlier studies have shown that females have higher IgG EV antibody levels than males, associations between IgA EV antibody levels and gender have not been demonstrated [9, 31]. The role of gender in susceptibility to IgA EV needs further research.

Another important finding of this study is the association between GADA positivity and elevated IgA EV levels in T1D. We believe that this finding confirms once again the hypothesis that EV infections may be involved in the development of immune reactions against 65 kDa glutamic acid decarboxylase, an antigenic target of GADA [32]. We also showed association between EV IgG and ZnT8A; however, since ZnT8A has been described quite recently, earlier studies have not measured it [28, 29]. According to a previous study, children with AAB had considerably higher abundance of EV (coxsackie A) species in the gut compared with controls, while there were no differences in virus positivity between them [29]. Similarly, we showed elevated levels of IgA EV in participants positive for multiple AAB in our study.

The current study shows that in the T1D group, the risk for elevated IgG EV antibody level increases significantly with age. This could be explained by the fact that exposure to different viruses, including different strains of EV, increases with age [33]. EV infections occur most often in childhood, but adults can be also infected, although clinical presentations in this case may be milder and less identifiable than those in children [34].

Earlier studies have demonstrated associations between HLA and EV antibody [35, 36]. To reduce flaws due to HLA genotype disparities between the control and participants with T1D in the present study, we stratified the study groups according to three principal genotypes: HLA genotypes with increased, neutral, and decreased risk for T1D. The principle of such grouping was drawn from a study by Hermann et al. [37]. In our study, however, due to the limited number of study subjects, we used three groups instead of six in the original study.

We failed to detect associations between EV antibodies and PTPN22 and IFIH1 gene polymorphism, which have been shown to be connected with the immune system's reactivity against viruses. Some previous studies have demonstrated associations of IFIH1 [38] and PTPN22 with EV infections [39]. The discrepancy between our results and those of other investigators might be explained by general genetic differences between the target populations.

Although the major merit of this study is a match between cases and controls by age, gender, and HLA-DR/DQ genotype risk groups for T1D, it has some limitations that need to be considered. The first limitation of the study is that the number of participants was relatively small: 62 participants with T1D and the same number of controls. However, we must emphasize that all participants with T1D were recently diagnosed cases, which provides an excellent opportunity to demonstrate, through the presence of IgA antibodies, the role of EV in T1D. Another limitation is the use of a synthetic common EV peptide instead of different EV antigens in individual antibody assays. However, because of the great number of different EVs, which have so far been demonstrated to be associated with T1D [40], our approach might even be more justified. This is also a strength of our study, as it allows comparing the summation of exposures to different EVs for controls and for participants with T1D.

## 5. Conclusions

Our study based on age-, gender-, and HLA-matched patients and controls showed that, like childhood-onset T1D, development of adulthood T1D might be associated with EV infections, as shown by the presence of IgA antibodies against EV. These antibodies may be associated with lasting mucosal EV infections rather than with recent acute EV infections, since IgM antibodies against EV were rarely detected in participants with T1D in this study. In addition, we found the association of GADA- and multiple AAB positivity with elevated IgA EV levels among participants with T1D. To confirm the association between adult-onset T1D and EV infection, direct virological studies are needed.

## Figures and Tables

**Table 1:** Characteristics of the study populations.

Characteristics	Control (*n* = 62)	T1D (*n* = 62)	*p* values
Gender (female/male)	29 (46.8%)	29 (46.8%)	1
Age at the time of study (y)	13.9 (8.0–21.9)	13.9 (7.9–22.4)	0.9741
Age groups			1
0–18.9 years	36 (58.1%)	36 (58.1%)	
19–35.9 years	26 (41.9%)	26 (41.9%)	
HLA genotype risk group			1
Increased risk	25 (40.3%)	25 (40.3%)	
Neutral	23 (37.1%)	23 (37.1%)	
Decreased risk	14 (22.6%)	14 (22.6%)	
PTPN22 (rs2476601)			0.1692
CC	44 (71.0%)	37 (59.7%)	
CT	18 (29.0%)	22 (35.5%)	
TT	0 (0.0%)	3 (4.8%)	
IFIH1 (rs1990760)			0.1599
AA	20 (32.3%)	30 (48.4%)	
AG	35 (56.5%)	25 (40.3%)	
GG	7 (11.3%)	7 (11.3%)	
Autoantibodies (AAB)			
GADA	0 (0.0%)	52 (83.9%)	
IA2A	0 (0.0%)	35 (56.5%)	
ZnT8A	0 (0.0%)	40 (64.5%)	
Positive for 1 AAB	0 (0.0%)	14 (22.6%)	
Positive for 2 AAB	0 (0.0%)	19 (30.6%)	
Positive for 3 AAB	0 (0.0%)	25 (40.3%)	
Ketoacidosis	—	24^a^ (38.7%)	
Ketonuria	—	45^b^ (72.6%)	
Weight loss	—	40^c^ (64.5%)	
Season of sampling			0.4625
September-February	40 (64.5%)	35 (56.5%)	
March-August	22 (35.5%)	27 (43.5%)	

The subjects were matched by gender, age, and HLA. Due to the high variability of HLA genotypes in the study groups, three main HLA genotype risk groups were distinguished. Nonparametric data are presented as the number of persons in the group and a percentage in a column. Numerical data are presented as the median and interquartile range (IQR). Differences in the characteristics between controls and participants with type 1 diabetes (T1D) were calculated with the chi-square test, Fisher's exact test, or Mann-Whitney *U* test, and *p* values are presented. ^a^Ketoacidosis data missing: *n* = 1 participant with T1D. ^b^Ketonuria data missing: *n* = 5 participants with T1D. ^c^Weight loss data missing: *n* = 1 participant with T1D.

**Table 2:** Prevalence of antibodies to enteroviruses (EV) in participants with type 1 diabetes (T1D) and controls.

Antibodies	Control		T1D		Matched OR^∗^	
	*n*	% (95% CI)	*n*	% (95% CI)	(95% CI)	*p* value
IgA EV	7/62	11.3 (5.0–22.5)	29/62	46.8 (34.2–59.8)	**8.33 (2.52**–**27.6)**	**0.0005**
IgG EV	29/62	46.8 (34.2–59.8)	30/62	48.4 (35.7–61.3)	1.07 (0.52–2.22)	0.8530
IgM EV	1/57	1.8 (0.09–10.6)	3/57	5.2 (1.4–15.5)	3.00 (0.31-28.8)	0.3410

Data are provided as the number of EV-positive persons and percentage with the 95% confidence interval (CI). ^∗^Conditional logistic regression model: matched odds ratio for gender, age, and HLA-DR/DQ genotype risk groups for T1D with the 95% confidence interval (CI). *p* values ≤ 0.05 are in bold.

**Table 3:** Prevalence of IgA, IgG, and IgM antibodies to enteroviruses (EV) in the study population by the different age groups.

Age groups	Control		T1D		*p* value	All	
	*n*	% (95% CI)	*n*	% (95% CI)		*n*	% (95% CI)
IgA EV antibodies							
0–18.9 y	5/36	13.9 (5.2–30.3)	16/36	44.4 (28.3–61.7)	**0.0098**	21/72	29.2 (19.3–41.2)
19–35.9 y	2/26	7.7 (1.3–26.6)	13/26	50.0 (32.1–67.9)	**0.0055**	15/52	28.8 (17.5–43.3)
IgG EV antibodies							
0–18.9 y	18/36	50.0 (34.5–65.5)	15/36	41.7 (26.0–59.1)	NS	33/72	45.8 (34.1–57.9)
19–35.9 y	11/26	42.3 (24.0–62.8)	15/26	57.7 (37.2–76.0)	NS	26/52	50.0 (36.9–63.1)
IgM EV antibodies							
0–18.9 y	1/36	2.8 (0.1–16.2)	2/36	5.6 (9.7–20.0)	NS	3/72	4.2 (1.1–12.5)
19–35.9 y	0/26		1/26	3.8 (0.2–21.6)	NS	1/52	1.9 (0.1–11.6)

Data are presented as the number of EV antibody-positive persons and percentage with the 95% confidence interval (CI) in a row. Cochran-Mantel-Haenszel test, matched for gender, age, and HLA-DR/DQ genotype risk groups for type 1 diabetes (T1D). *p* values ≤ 0.05 are in bold. NS: not significant.

## Data Availability

The datasets generated during and/or analysed during the current study are available from the corresponding author on reasonable request.

## Supplementary Materials

Supplementary Materials Supplementary Table 1: median IgA and IgG EV levels with the interquartile range (IQR) among autoantibodies (AAB).
