# Supplementary material for: IgA-Type Enterovirus Antibodies Are Increased among Adults and Children with Recently Diagnosed Type 1 Diabetes
Source: Biomed Res Int. 2022 Jul 31;2022:7603062. doi: 10.1155/2022/7603062 (PMC9357813; doi:10.1155/2022/7603062)
Supplement: Supplementary Materials — Supplementary Table 1: median IgA and IgG EV levels with the interquartile range (IQR) among autoantibodies (AAB). [file 7603062.f1.docx]

Supplementary Table 1. Median IgA and IgG EV levels with the interquartile range (IQR) among autoantibodies (AAB).

| Autoantibodies | Median IgA EV level (IQR) | Median IgG EV level (IQR) |
| --- | --- | --- |
| GADA: |  |  |
| Positivity | 13.5 (5.97–32.5) | 19.1 (5.0–47.7) |
| Negativity | 2.7 (0.0–16.0) | 7.9 (3.9–14.4) |
| IA2A: |  |  |
| Positivity | 17.6 (5.2–40.3) | 12.4 (4.9–40.9) |
| Negativity | 10.5 (2.7–19.8) | 12.8 (4.4–42.8) |
| ZnT8A: |  |  |
| Positivity | 10.5 (3.2–34.1) | 11.9 (4.5–35.3) |
| Negativity | 16.4 (5.9–27.0) | 13.9 (5.4–51.0) |
| AAB count: |  |  |
| 0 | 5.2 (0.0–12.3) | 9.2 (2.6–15.8) |
| 1 | 7.8 (2.5–20.7) | 10.0 (5.6 –46.0) |
| 2 | 17.6 (7.15–31.0) | 29.5 (3.8–59.1) |
| 3 | 10.2 (5.1–50.5) | 11.3 (4.6–34.1) |
| AAB: |  |  |
| <2 AAB positivity | 7.8 (2.0–17.8) | 10.0 (5.4–29.4) |
| ≥2 AAB positivity | 14.0 (5.8–34.1) | 19.1 (4.5–42.7) |
